# The Fabrication of Amino Acid Incorporated Nanoflowers with Intrinsic Peroxidase-like Activity and Its Application for Efficiently Determining Glutathione with TMB Radical Cation as Indicator

**DOI:** 10.3390/mi12091099

**Published:** 2021-09-12

**Authors:** Ning Jiang, Chuang Zhang, Meng Li, Shuai Li, Zhili Hao, Zhengqiang Li, Zhuofu Wu, Chen Li

**Affiliations:** 1 Key Laboratory of Zoonosis Research, Ministry of Education, Institute of Zoonosis, College of Veterinary Medicine, Jilin University, Changchun 130062, China; jiangning19@mails.jlu.edu.cn (N.J.); H1455421950@163.com (Z.H.); 2 Key Laboratory for Molecular Enzymology and Engineering of the Ministry of Education, College of Life Sciences, Jilin University, Changchun 130012, China; zhangchuang19@mails.jlu.edu.cn (C.Z.); ls2012@jlu.edu.cn (S.L.); lzq@jlu.edu.cn (Z.L.); 3 State Key Laboratory of Supramolecular Structure and Materials, College of Chemistry, Jilin University, Changchun 130012, China; lmeng17@mails.jlu.edu.cn; 4 Key Laboratory of Straw Biology and Utilization, The Ministry of Education, College of Life Science, Jilin Agricultural University, Changchun 130118, China

**Keywords:** amino acid-incorporated nanoflowers, glutathione, peroxidase-like activity

## Abstract

The assessment of glutathione (GSH) levels is associated with early diagnostics and pathological analysis for various disorders. Among all kinds of techniques for detecting GSH, the colorimetric assay relying on the oxidation of 3,3′,5,5′-tetramethylbenzidine (TMB) catalyzed by many nanomaterials with peroxidase-like activity attracts increasing attention owing to its outstanding merits, such as high sensitivity and high selectivity. However, the aggregation between the nanomaterials severely hinders the entrance of TMB into the “active site” of these peroxidase mimics. To address this problem, the D-amino acid incorporated nanoflowers possessing peroxidase-like activity with a diameter of 10–15 μm, TMB and H_2_O_2_ were employed to establish the detection system for determining the level of glutathione. The larger diameter size of the hybrid nanoflowers substantially averts the aggregation between them. The results confirm that the hybrid nanoflowers detection system presents a low limit of detection, wide linear range, perfect selectivity, good storage stability and desired operational stability for the detection of GSH relying on the intrinsic peroxidase-like activity and favorable mechanical stability of the hybrid nanoflowers, indicating that the hybrid nanoflowers detection system has tremendous application potential in clinical diagnosis and treatment.

## 1. Introduction

In general, the reduction of glutathione (GSH; g- l-glutamyl-l-cysteinylglycine) levels is associated with many human diseases, such as neurodegenerative diseases, cystic fibrosis (CF), acquired immune deficiency syndrome (AIDS), liver disease and aging, etc. [1,2,3,4,5,6,7]. In contrast, it is well documented that elevated GSH levels can be identified in many types of tumor cells, which facilitates the removal and detoxification of carcinogens during cancer development, even if such an elevated state may also conversely improve the resistance to chemotherapeutic drugs [8]. Hence, the determination of the level of GSH is important for accomplishing the early diagnostics and pathological analysis for these disorders.

So far, there are numerous methods proposed for determining the fluctuation of GSH levels for early diagnosis of the relevant diseases and the evaluation of the progression of these disorders. These methods contain the high-performance liquid chromatography approaches combined with different detection systems including UV absorbance, fluorometric detection, electrochemical detection, mass spectrometry, the capillary electrophoresis system coupled with the above-mentioned different detection systems, magnetic resonance spectroscopy and on-capillary surface-enhanced Raman spectroscopy (SERS) method [9,10,11,12,13,14,15,16,17,18].

Although these methods present the ideal sensitivity and selectivity for assaying the level of GSH in human cells or tissues, the application of a fluorometric assay suffers from the difficulties caused by the necessity for the derivatization procedures of the samples before measurement, the fact that the utilization of an electrochemical detector needs an operating electrochemical cell with a higher applied oxidation potential, resulting in its shortened life span, and high-performance liquid chromatography, capillary electrophoresis, mass spectrometry, magnetic resonance spectroscopy and surface-enhanced Raman spectroscopy require the use of the expensive, sophisticated and specialized apparatus as well as the requirement for experienced technicians, which severely confines the applications of these techniques in clinical chemistry [19,20,21,22,23,24,25,26,27,28,29,30,31,32,33]. Conversely, the colorimetric assay has received continuous attention as an attractive alternative strategy because it is a simple, convenient, sensitive and accurate route to accomplish the assessment.

Though the recycling assays comprised by the oxidation of GSH catalyzed by the sulfhydryl reagent and the reduction of GSSG catalyzed by glutathione reductase were implemented to assess the level of GSH in whole blood, plasma, serum, lung lavage fluid, cerebrospinal fluid, urine, tissues and cell extracts via spectrophotometer reader assay in practice, the application of the commercial GSH kits based on this principle still face the issues of the higher cost and the inactivity of the enzyme used [34]. In practical applications, the high cost of purification and rigorous storage conditions largely limits the utilization of natural enzymes [35]. Therefore, numerous efforts have been devoted to solving these problems by developing novel enzyme mimics to replace the natural enzyme [36]. To our knowledge, several nanostructures, such as gold nanoclusters, Co_3_O_4_ nanoparticles, gold nanoparticles, FeS_2_ nanoparticles, or Si doped CoO nanorods possessing instinct peroxidase-like activity were fabricated to detect GSH owing to its greater resistance to adverse surroundings and cost-effectiveness [37,38,39,40,41]. The oxidation of 3,3′,5,5′-tetramethylbenzidine (TMB) catalyzed by the mimetic peroxidases can be suppressed by GSH, accompanied by a sharp decrease of the absorbance at 652 nm. However, the above mentioned nanostructures are inevitably apt to aggregate during assay development, resulting in poor catalytic performance for the measurement of GSH [42].

In 2016, amino acid incorporated nanoflowers were constructed by our group relying on the self-assembly between the amino acids and copper phosphate, and the resulting amino acid incorporated nanoflowers unexpectedly give peroxidase-like activity based on the catalytic principle of Fenton’s like reagents and successfully fulfill the conversion of ABTS to ABTS radical cation (oxidized ABTS) [43]. Because the amino acid incorporated nanoflowers have a flower-like porous structure with diameters in the range of 10–15 µm, the aggregation between the nanoflowers can be avoided. It is reported that, in the Ellman method, a typical route for GSH detection, the absorption maximum of the color indicator 3-carboxy-4-nitrobenzenethiolate anion (TNB^-^) at 412 nm coincides with the Soret band of hemoglobin at 410 nm, which significantly weakens the assay sensitivity for GSH detection in the analysis of the whole blood or erythrocyte [44]. Considering that ABTS radical cation produced by peroxidase has an absorption maximum at 414 nm, the ABTS method should not allow the determination of GSH levels in the whole blood or erythrocyte owing to the interference with hemoglobin absorption [45]. In contrast, since TMB radical cation generated by peroxidase exhibits the maximum absorbance at 652 nm, which is far from the hemoglobin Soret band, TMB was chosen as the chromogen to reflect the concentration of GSH in this work [46].

The amino acid was selected as an organic component to direct the synthesis of organic-inorganic hybrid nanoflowers. It is expected that amino acid incorporated nanoflowers could take on superior sensitivity and perfect operational stability for GSH detection. The scanning electron microscope (SEM), Raman spectroscopy and Diffuse Reflectance UV–vis spectroscopy were applied to characterize the nanoflowers, and then the enzymatic assay and kinetic analysis of the nanoflowers were performed to evaluate their enzymatic properties. Subsequently, the GSH detection assay was accomplished depending on the peroxidase-mimicking activity of the nanoflowers for ascertaining the detection limit, linear range and tsensitivity of this detection system. Finally, the storage stability and reusability were carried out to evaluate the potential value of this detection system in practical applications.

## 2. Materials and Methods

### 2.1. Materials

d-Alanine, d-Arginine, d-Asparticacid, d-Asparagine, d-Glutamicacid, d-Histidine, d-Isoleucine, d-Leucine, d-Lysine, d-Methionine, d-Phenylalanine, d-Proline, d-Serine, d-Threonine, d-Tryptophan, d-Tyrosine, d-Valine, KBr (spectral grade), bovine serum albumin (BSA), glutathione and 3,3′,5,5′-tetramethylbenzidine (TMB) were purchased from Sigma-Aldrich Chemical Co. (St. Louis, MO, USA). d-Cystine and d-Glutarnine were purchased from Aladdin Reagent Co, Ltd. (Shanghai, China). All other chemicals and reagents were of analytical grade. All aqueous solutions were prepared with Milli-Q water.

### 2.2. Preparation of Amino Acid-Incorporated Nanoflowers

The d-amino acid-incorporated nanoflowers were prepared as reported previously [40]. Briefly, amino acid powder (60 mg) was dissolved in 3 L of PBS solution (10 mM, pH 7.4), and then 20 mL of CuSO_4_ solution (120 mM) was added into the above-mentioned mixture, followed by the incubation at room temperature for three days. The resulting blue precipitate could be observed at the bottom of the beaker. The precipitate was obtained through centrifugation (12,000 rpm for 20 min) and rinsed with deionized water three times. Finally, the cleaned product was stored in the refrigerator at 4 °C until use.

### 2.3. Characterization of Amino Acid-Incorporated Nanoflowers

Scanning electron microscopy investigation was carried out using a JSM-IT500A electron microscope (JEOL, Tokyo, Japan) under the condition of 30 kV acceleration voltages. The assessment of Raman spectra was performed by JY-T64000 (HORIBA Jobin Yvon, Paris, France) equipped with a 532 nm laser. The instrument is equipped with a microscope with a focal spot size in the range of a few micrometers. After pretreating for 1 h under vacuum, the nanoflowers were characterized by a Lambda 1050+ (PerkinElmer, Waltham, MA, USA) Diffuse reflectance UV-vis Spectrometer (DRUVS) using BaSO4 as a reference to determine its UV-vis absorption spectra.

### 2.4. Analysis of Enzyme Assay

#### 2.4.1. The Assessment of Peroxidase-like Activity

The peroxidase-like activity of the hybrid nanoflowers was measured based on TMB (3,3′,5,5′-tetramethylbenzidine) method [43]. The reaction system comprised of d-isoleucine (ILE) incorporated nanoflower (50 μg/mL), H_2_O_2_ (25 mM) and TMB (2 mM) was incubated at 25 °C for 5 min. The absorbance at 652 nm was then recorded by a UV–vis spectrophotometer. The effect of pH on the peroxidase-like activity of the samples was studied in the range 2.0~12.0. The effect of temperature on the peroxidase-like activity of the samples was investigated ranging from 15–65 °C. The assay procedures were similar to the protocol as above described.

#### 2.4.2. Kinetic Parameters

*K*_m_ and V_max_ values for the peroxidase-like activity of the hybrid nanoflowers were calculated from a Lineweaver–Burk plot through surveying the initial rates of the reactions using various H_2_O_2_ concentrations ranging from 0.01 to 25.0 mM at 2 mM TMB or various different TMB concentrations in the range of 0.0625 to 4.0 mM at 25 mM H_2_O_2_ according to the previous report [47].

### 2.5. The Detection of GSH

#### 2.5.1. Detection of H_2_O_2_

The linearity and limit of detection for the detection of H_2_O_2_ were conducted with the hybrid nanoflowers (50 μg/mL), TMB (2 mM), and serial concentration of H_2_O_2_ (10–800 μM) in an acetic acid-sodium acetate buffer (pH 4.0). The detection system was incubated in the dark for 10 min, followed by centrifuging at 12,000 rpm for three min. The absorbance of the supernatant at 652 nm was monitored by a UV-Vis spectrophotometer. The absorbance value of the supernatant at 652 nm stands for the level of peroxidase-like activity of the nanoflowers.

#### 2.5.2. Detection of GSH

The linearity and limit of detection for the detection of GSH were conducted as follows. The hybrid nanoflowers (50 μg/mL) and H_2_O_2_ (25 mM) were mixed with TMB (2 mM) in an acetic acid-sodium acetate buffer (pH 4.0). The mixture was incubated in the dark for 10 min. Subsequently, GSH with different concentrations (0.5–50 μM) was added to the mixture. The supernatant was separated from the mixture by centrifuging at 12,000 rpm for three min, and then the absorbance of the supernatant was recorded using a UV-Vis spectrophotometer. The change in the absorbance at 652 nm in the presence or absence of GSH was calculated and defined as ΔA. The value of ΔA stands for the detection capability of the detection system in this work.

#### 2.5.3. The Interference Experiment

The interference experiment for GSH detection was accomplished by comparing the changes in the absorbance of oxide TMB at 652 nm caused by the addition of GSH in the presence of various interference species, respectively. The various interference species included 1 mM of bovine serum albumin, Zn^2+^, Ca^2+^, Na^+^, Mg^2+^, K^+^, NH_4_^+^, glucose, d-proline, d-cystine, d-arginine, d-lysine, d-alanine, d-leucine, d-methionine and d-histidine. The assay procedure was implemented according to the description in Section 2.5.2.

#### 2.5.4. Stability and Reusability of the Hybrid Nanoflowers

The hybrid nanoflowers exposed in the air at 25 °C were taken each day and then employed for detecting GSH. The performance of the hybrid nanoflowers for detecting GSH during continuous recycling use was assessed. The assay procedure was carried out based on the description in Section 2.5.2.

## 3. Results and Discussion

### 3.1. SEM Images of the Hybrid Nanoflowers

**Figure 1:**
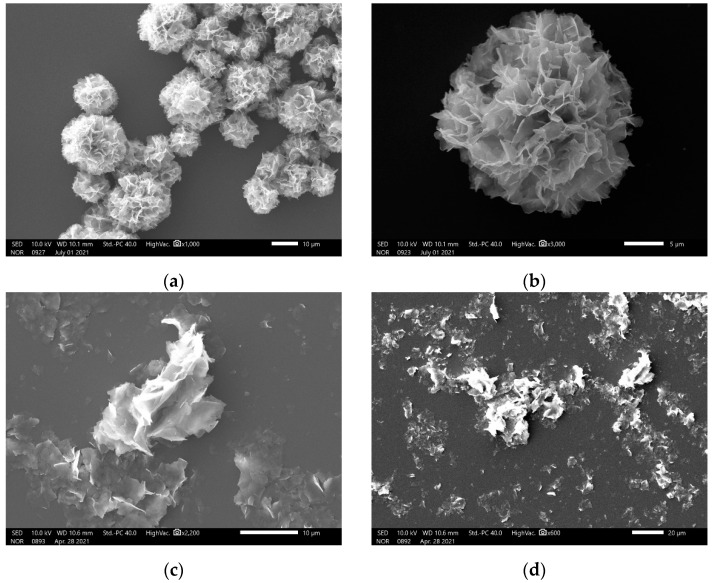
SEM images of d-isoleucine-incorporated nanoflowers (**a**), a single nanoflower (**b**) and Cu_3_(PO_4_)_2_ matrices without d-isoleucine (**c**,**d**).

The SEM images indicate that the nanomaterial with hierarchical structures can be formed in the presence of ILE, and that this kind of nanomaterial has flower-like nanostructures and a size of about 10–15 μm (Figure 1a,b). When 18 other kinds of amino acids were added to the synthesis system, the same nanomaterials could be seen (Figure S1). In contrast, the results show that the disorder fragment can be obtained in the absence of amino acids (Figure 1c,d). These results suggest that amino acids play a critical role in directing the synthesis of flower-like nanostructures. Furthermore, the slight change in the morphology of the flower-like nanostructures occurs with the change of the amino acid employed, suggesting that the side chain of amino acid is involved in the formation of the coordination bonds between the amino acid and Cu ion, which is in accordance with the synthesis principle of the protein incorporated nanoflowers [48]. It could be found that ILE incorporated nanoflowers have a higher intrinsic peroxidase-like activity as compared with other amino acid incorporated nanoflowers in the pre-experiment. Hence, ILE incorporated nanoflowers were employed in the following experiment.

### 3.2. Raman Spectrum

**Figure 2:**
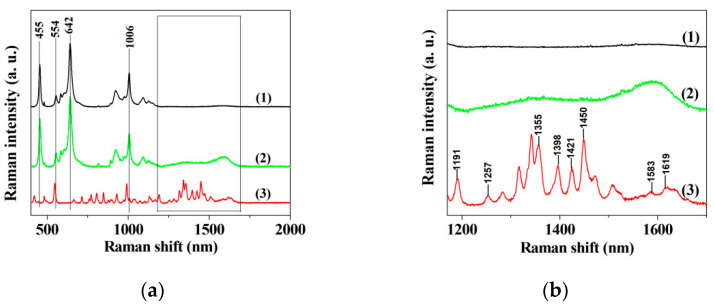
(**a**) Raman spectrum of Cu_3_(PO_4_)_2_ crystal without ILE (1), the hybrid nanoflowers (2) and ILE (3); (**b**) The enlarged view of the Raman spectra in the range of 1170–1700 cm^−1^.

The Raman spectra in Cu_3_(PO_4_)_2_ matrices without ILE, ILE-incorporated nanoflower and ILE are shown in Figure 2a,b. The three bands at 455 cm^−1^, 642 cm^−1^ and 1006 cm^−1^ in curve 1 and 2 of Figure 2a are assigned to the PO3 symmetric bending vibration, the PO3 out-of-plane bending vibrations and the PO3 antisymmetric stretching vibration, respectively [49]. The three bands arise from the contribution of Cu_3_(PO_4_)_2_. Furthermore, the bands at 1257 cm^−1^, 1355 cm^−1^, 1398 cm^−1^, 1421 cm^−1^, 1450 cm^−1^, 1583 cm^−1^ and 1619 cm^−1^ can be observed at curve 3 of Figure 2a,b, and these bands are assigned to the characteristic absorption of isoleucine [50]. No characteristic bands of isoleucine can be seen at curve 1 of Figure 2a,b, and these bands become broaden at curve 2 of Figure 2a,b, probably attributing to the interaction between Cu_3_(PO_4_)_2_ matrices and isoleucine. It is speculated from curve 2 of Figure 2a that the ILE incorporated nanoflowers give the characteristic absorption of both Cu_3_(PO_4_)_2_ and isoleucine, verifying that ILE is present in the Cu_3_(PO_4_)_2_ nanoflowers.

### 3.3. Diffuse Reflectance UV–Vis Spectra of the Hybrid Nanoflowers

**Figure 3:**
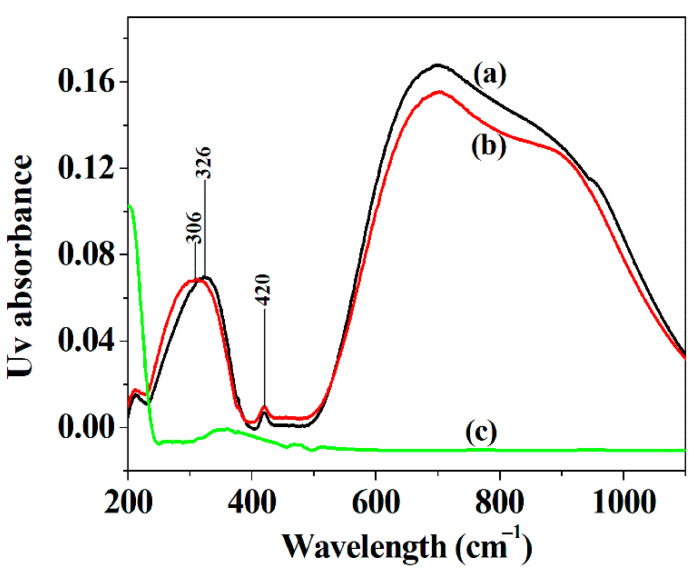
Cu_3_(PO_4_)_2_ crystal without ILE (**a**), the hybrid nanoflowers (**b**) ILE (**c**).

As shown in Figure 3, the hybrid nanoflowers exhibit a narrower absorption range in the wavelength range of 500–1200 cm^−1^ in Diffuse Reflectance UV–vis spectra as compared with Cu_3_(PO_4_)_2_ crystal, and the blue shift of the peak of Cu_3_(PO_4_)_2_ component in the hybrid nanoflowers in the wavelength range 200–400 cm^−1^ happens after isoleucine is added to the synthesis route in comparison with Cu_3_(PO_4_)_2_ crystal (curve a and curve b). These results show that the incorporation of isoleucine may affect the absorption of the spectrum of two copper sites in the copper phosphate component of the hybrid material [51].

### 3.4. The Peroxidase-like Activity of the Hybrid Nanoflowers

**Figure 4:**
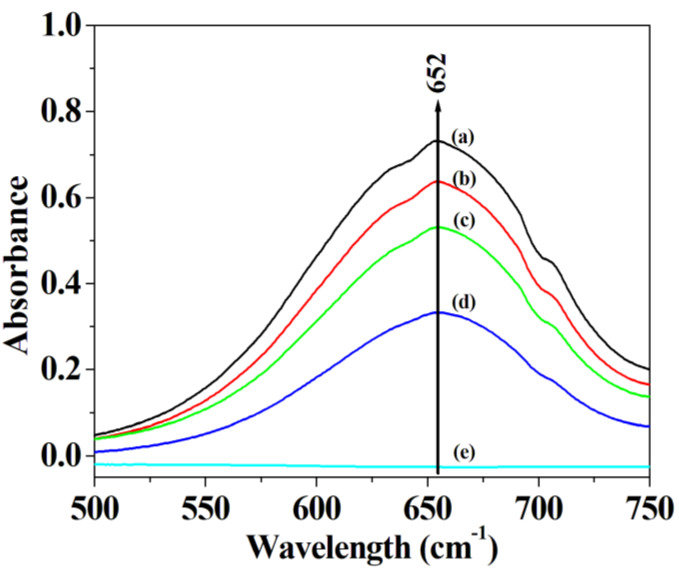
Effect of the concentration of the nanoflowers on its peroxidase-like activity: (**a**) 20 μg/mL of the nanoflowers, (**b**) 15 μg/mL of the nanoflowers, (**c**) 10 μg/mL of the nanoflowers and (**d**) 5 μg/mL of the nanoflowers and (**e**) no nanoflowers.

To investigate the mimetic activity of the hybrid material, the different amount of hybrid nanoflowers was employed to oxide TMB with the help of H_2_O_2_. The experimental data demonstrate that the absorbance of oxidized TMB at 652 nm significantly increase after adding hybrid nanoflowers, and that the increasing rise of the absorbance at 652 nm occurs with the increase of the amount of hybrid nanoflowers employed (Figure 4), which is similar to Wang et al.’s results [47]. They believed that the remarkable elevation in UV absorbance of oxidized TMB shall be attributed to the contribution of peroxidase-like activity offered by the nanomaterials through the catalytic mechanism of Fenton’s like reagents.

### 3.5. The Influence of Temperature and pH on the Mimetic Activity

**Figure 5:**
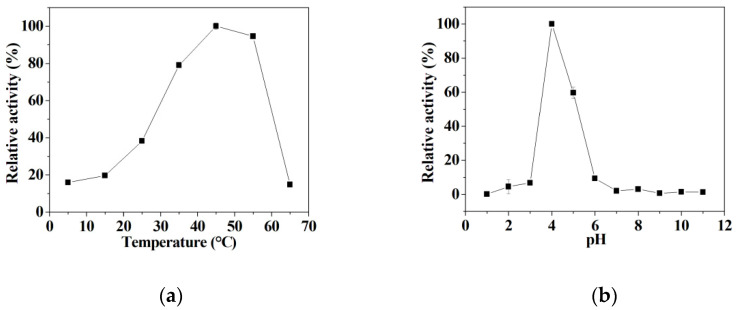
(**a**) The effect of temperature on the peroxidase-like activity of the nanoflowers; (**b**) The effect of pH on the peroxidase-like activity of the nanoflowers.

It is can be seen that the peroxidase-like activity of hybrid nanoflowers gradually increases with the increase of temperatures in the range of 5~45 °C, and then drops quickly with the further increase of temperatures ranging from 45 °C to 65 °C (Figure 5a). In addition, it is shown that the peroxidase-like activity significantly rises in the pH range of 3.0~4.5, and then sharply declines with the further change of pH ranging from 4.5~6.0 (Figure 5b). The hybrid nanoflowers present the maximum mimetic activity at pH 4.5. Unfortunately, the hybrid nanoflowers only give negligible peroxidase mimetic activity in the pH range of 1.0~3.0 and pH range of 6.0~11.0 (Figure 5b); this is attributable to the inactivation of the enzyme under drastic conditions [52]. In the following study, the detection system was performed at 45 °C in a pH 4.5 buffer to exert the peroxidase-like activity of the nanoflowers.

### 3.6. Kinetic Analysis

**Figure 6:**
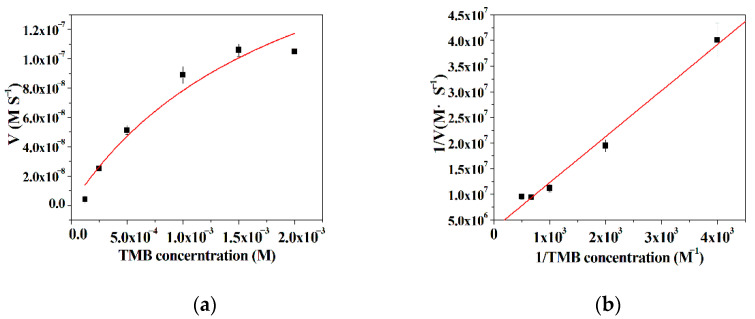
(**a**) The effect of 3,3′,5,5′-tetramethylbenzidine (TMB) concentration on the peroxidase-like activity of the nanoflowers; (**b**) Lineweaver–Burk plots of the reaction velocity of the nanoflowers as a function of TMB concentration.

**Figure 7:**
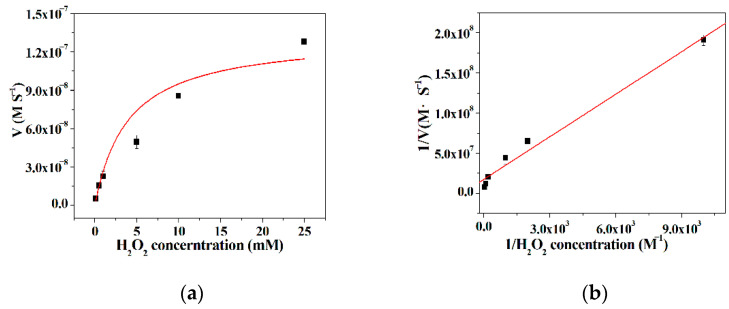
(**a**) The effect of H_2_O_2_ concentration on the peroxidase-like activity of the nanoflowers; (**b**) Lineweaver–Burk plots of the reaction velocity of the nanoflowers as a function of H_2_O_2_ concentration.

The kinetic parameters were obtained by surveying the initial rate of the TMB oxidation reaction, as the concentration of either TMB or H_2_O_2_ was kept constant while the concentration of the other substrate varied. It is can be found from Figure 6a and Figure 7a that there is a good linear relationship between the initial rate of the TMB oxidation reaction and the substrates as a low concentration substrate is used, and then the initial rate progressively reaches a plateau with the extension of the concentration of the substrates. These results reveal that the kinetic behavior of the hybrid nanoflowers using TMB or H_2_O_2_ as the substrates closely resembles the example of the native enzymes [53]. According to the double reciprocal plots in Figure 6b and Figure 7b, the kinetic constants *K*_m_ and V_max_ can be acquired and presented in Table 1. From Table 1, the *K*m value of the hybrid nanoflowers is 6.03 times higher than that of the horseradish peroxidase (HRP) as the concentration of TMB varies, while the *K*_m_ value of the hybrid nanoflowers is nearly three times lower than the case of the native counterpart when the concentration of H_2_O_2_ varies. The decreased *K*_m_ value for the enzymatic mimic means the increased affinity, which suggests that the hybrid nanoflowers exhibit more “active sites” on its surface in comparison with HRP possessing one active site per enzyme molecule [54]. Moreover, the V_max_ value of the hybrid nanoflowers is about three times lower than that of HRP as the concentration of TMB changes, and the hybrid nanoflowers give 1.5 fold decreases in V_max_ value compared with HRP.

### 3.7. The Detection of GSH

In our previous work, it is suggested that amino acid incorporated nanoflowers react with H_2_O_2_ to produce the highly reactive hydroxyl radical via the copper-redox cycle by its peroxidase-like activity, and that the resulting hydroxyl radical triggers the oxidization of ABTS (Formulas (1) and (2)) [43]. In this work, the presence of Cu^2+^ ion and Cu^+^ ion in the hybrid nanoflowers was confirmed by the XPS spectrum, and the ratio between Cu^2+^ ion to Cu^+^ ion is 1:0.374 (Figure S2 in Supplementary Information). According to the published reports, several peroxidase mimics generate hydroxyl radical by decomposing H_2_O_2_ to accomplish the conversion of TMB to TMB radical cation (oxidized TMB) relying on its peroxidase-like activity (Formula (3)) [40,41,55]. Therefore, the amino acid incorporated nanoflowers with intrinsic peroxidase-like activity were chosen as a peroxidase mimic to achieve the oxidation of TMB in this experiment. Moreover, it is well known that GSH can scavenge hydroxyl radicals accompanied by the conversion of GSH to GS• to resist the radiation damage to cells (Formula (4)) [56]. Several investigators also confirm that the sulfhydryl group offered by GSH can restore the TMB radical cation (oxidized TMB) to its original state by the hydrogen donation power of the sulfhydryl group (Formula (5)) [40,41,57,58,59]. Hence, it is speculated that the absorbance decline of TMB radical cation at 652 nm after adding GSH shall be attributed to the scavenging ability of GSH for hydroxyl radicals (Formula (4)) and the restoration ability of GSH for TMB radical cation (Formula (5)), which is the possible mechanism for the sensing of GSH using the hybrid nanoflowers detection system in this experiment.

Cu^2+^ + H_2_O_2_ → Cu^1+^ + HOO• + H^+^ (1)

Cu^1+^ + H_2_O_2_ → Cu^2+^ + •OH + OH^−^ (2)

•OH + TMB →TMB^+^• + H_2_O (3)

GSH + •OH → GS• + H_2_O (4)

GSH + TMB^+^• →TMB + GSSG (5)

**Figure 8:**
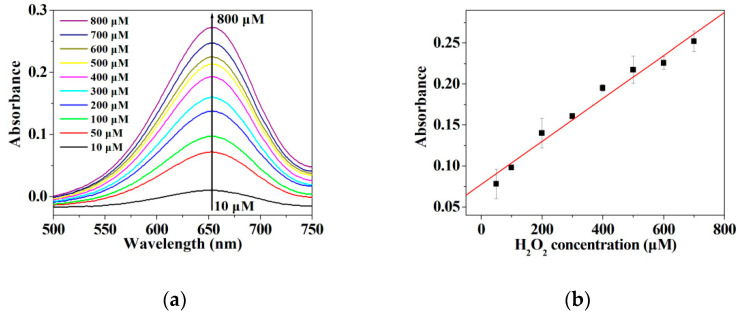
(**a**) UV-vis spectra of the oxTMB in the presence of different concentrations of H_2_O_2_; (**b**) Plots of the absorbance values at 652 nm vs. serial concentrations of H_2_O_2_.

**Figure 9:**
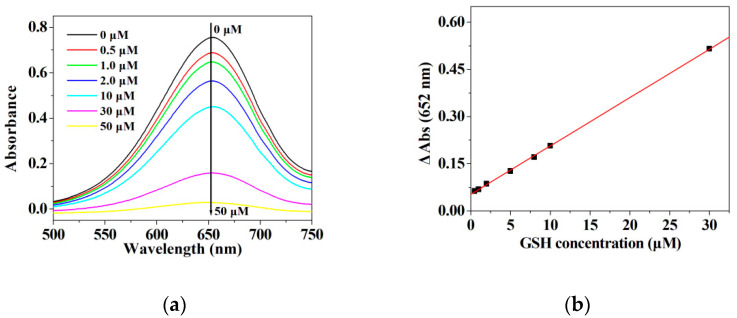
(**a**) UV-vis spectra of the oxTMB in the presence of different concentration of GSH; (**b**) Plots of the absorbance values at 652 nm vs. serial concentrations of GSH.

Given that other peroxidase mimics can promote the decomposition of H_2_O_2_ to generate •OH radicals which easily oxidize TMB to produce the oxidized TMB [40,41,55,60], it is necessary to determine the limit of detection and linear range for H_2_O_2_ when using the hybrid nanoflowers as the enzyme mimic for establishing the detection system in this work. As represented in Figure 8a,b, the absorbance at 652 nm is linearly correlated with the concentration of H_2_O_2_ ranging from 10 μM to 700 μM. The regression equation is determined to be A = 0.00026213 C+ 0.077451 with a correlation coefficient of 0.9868. As illustrated in Figure 9a,b, the absorbance of oxidized TMB is linearly correlated with the concentration of GSH in the range of 1–30 μM. The regression equation is ΔA = 0.01539 C_GSH_ + 0.05247 with a correlation coefficient of 0.9998. The limit of detection (LOD) was computed on the basis of 3σ/k, where σ and k are the relative standard deviation of ten parallel controlled measurements and the slope of the linear calibration plots, respectively. As for the detection of H_2_O_2_, the values of σ, k and LOD (H_2_O_2_) are 8.30662 × 10^−4^, 0.00026213 and 9.507 μM, respectively. As for the detection of GSH, the values of σ, k and LOD (H_2_O_2_) are 4.9 × 10^−4^, 0.01539 and 95.52 nM, respectively. The published results about GSH and H_2_O_2_ detection using the enzymatic-like activity of the peroxidase mimics are listed in Tables S1 and S2 [37,39,60,61,62,63,64,65,66,67,68,69,70,71,72,73,74,75,76]. It can be deduced from Tables S1 and S2 that the hybrid nanoflowers represent a satisfactory limit of detection as well as a wide linear range when detecting GSH and H_2_O_2_ as compared with other peroxidase mimics.

### 3.8. The Effect of Interferences Species on the Detection System

**Figure 10:**
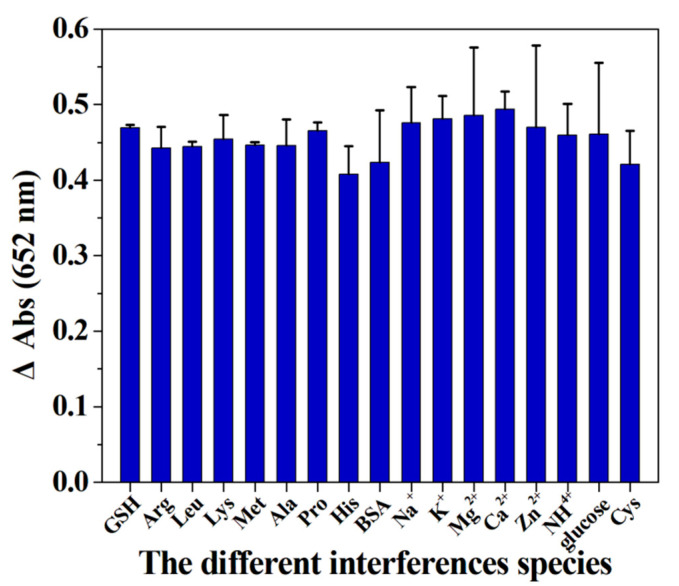
The detection capability of the detection system in the presence of various interferences species.

**Figure 11:**
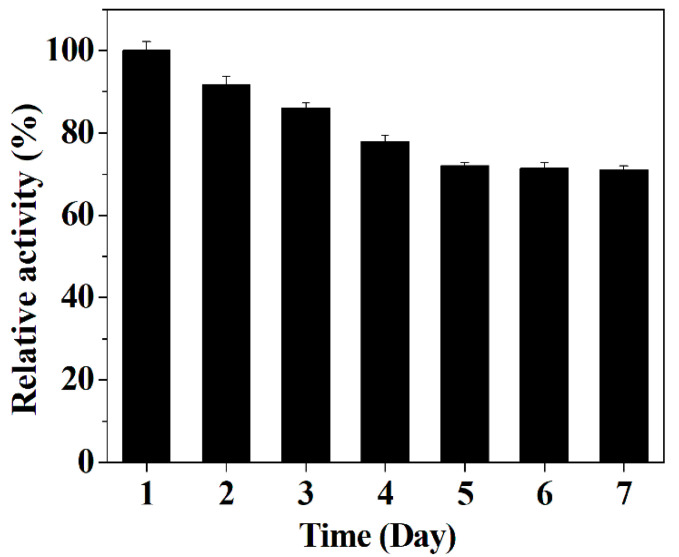
The stability of the hybrid nanoflowers detection system.

**Figure 12:**
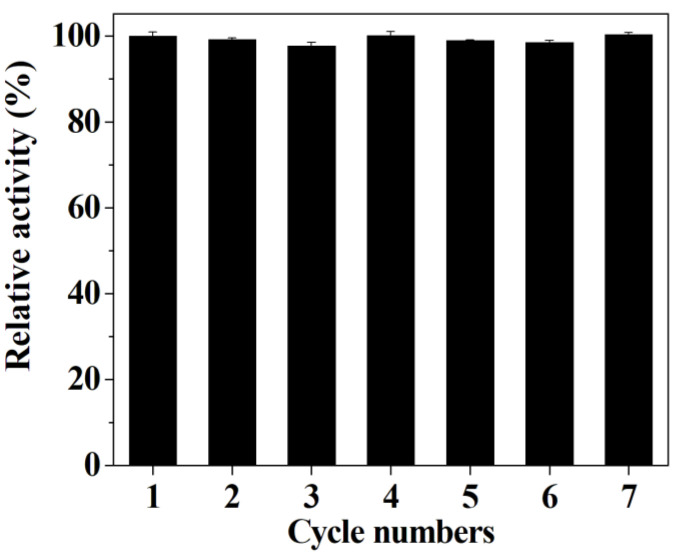
The reusability of the hybrid nanoflowers detection system.

In this work, the selectivity of the detection system towards GSH was investigated by adding various interference species. As illustrated in Figure 10, no obvious change in the value of ΔA can be observed after adding the above-mentioned interference substances, suggesting that the proposed detection system can be further employed to detect the level of GSH in biological samples. The results in Figure 11 verify that the hybrid nanoflower detection system still preserves about 70% of its initial detection capability after the hybrid nanoflowers had been exposed in air for seven days, and the data in Figure 12 reveal that the detection system almost maintains 100% of its initial detection capability even after sevens recycles. In short, although the limit of detection and linear range of the hybrid nanoflowers for determining GSH is not obviously superior to other peroxidase mimics, the easy large-scale preparation, mild synthesis route, superior stability, desired ligand grafting sites and lack of aggregation behavior of the hybrid nanoflowers make them a promising candidate for establishing the GSH detection system in the clinical diagnosis and treatment areas.

## 4. Conclusions

In this work, d-amino acid was used as a structure-directing agent to mediate the synthesis of organic-inorganic hybrid material with peroxidase-like activity. The SEM images reveal that the resulting hybrid material possesses flower-like nanostructures with a diameter of 10–15 μm. Subsequently, Raman spectra indicate that such hybrid material is made up of amino acid and Cu_3_(PO_4_)_2_ components. Hence, such flower-like organic-inorganic hybrid material was named amino acid-incorporated nanoflowers. Kinetic analysis manifests that the enzymatic behavior of amino acid-incorporated nanoflowers obeys the Michaelis-Menten model with TMB or H_2_O_2_ as the substrates. Most importantly, the hybrid nanoflowers are different from most nanomaterials with peroxidase-like activity, since their larger diameter size impedes the aggregation between them, which is helpful in exerting their peroxidase-like activity in clinical chemistry. Subsequently, the detection system composed of the hybrid nanoflowers, 3,3′,5,5′-tetramethylbenzidine and H_2_O_2_ was fabricated to determine glutathione. The dose-response curves prove that the hybrid nanoflowers detection system possess a wide linear range (10 μM–700 μM for H_2_O_2_, 1–30 μM for GSH) and low limit of detection (9.507 μM for H_2_O_2_, 95.52 nM for GSH) as compared with other detection approaches. No obvious change in the detection signal can be seen even after adding the most common interfering components to the sample solutions. Moreover, the hybrid nanoflowers not only maintain about 70% of initial detection capability even after having been exposed to air for seven days, but also preserve nearly 100% of initial detection capability after seven runs. All of the results suggest that the hybrid nanoflowers detection system is a promising strategy for detecting GSH in clinical diagnosis and treatment.

## Figures and Tables

**Table 1:** The comparison of kinetic constant between different catalysts.

Catalysts	Substrate	*K*_m_ (mM)	V_max_ (10^−8^ M S^−1^)
ILE incorporated nanoflowers	TMB	2.71	3.02
	H_2_O_2_	1.02	5.77
Native horseradish peroxidase	TMB	0.43	10
	H_2_O_2_	3.70	8.71

## Supplementary Materials

The following are available online at https://www.mdpi.com/article/10.3390/mi12091099/s1, Figure S1: The SEM images of the amino acid incorporated nanoflowers, Figure S2: XPS spectrum of ILE incorporated nanoflower, Table S1: The comparison in linear range and limit of detection between different detection systems for detecting H_2_O_2_, Table S2: The comparison in linear range and limit of detection between different detection systems for detecting GSH.
