# Supplementary material for: The Fabrication of Amino Acid Incorporated Nanoflowers with Intrinsic Peroxidase-like Activity and Its Application for Efficiently Determining Glutathione with TMB Radical Cation as Indicator
Source: Micromachines (Basel). 2021 Sep 12;12(9):1099. doi: 10.3390/mi12091099 (PMC8467630; doi:10.3390/mi12091099)
Supplement: Supplementary file 1 [file micromachines-12-01099-s001.zip › micromachines-1351030-supplementary.pdf]

The fabrication of amino acid incorporated nanoflowers with intrinsic peroxidase-like activity and its application for efficiently determining glutathione with TMB radical cation as indicator

Ning Jiang [1, 2#], Chuang zhang [2#], Meng Li [3], Shuai Li [2], Zhili Hao [1], Zhengqiang Li [2], Zhuofu Wu [4,*] and Chen Li [1,*]

1 Key Laboratory of Zoonosis Research, Ministry of Education, Institute of Zoonosis, College of Veterinary Medicine, Jilin University, Changchun 130062, China; jiangning19@mails.jlu.edu.cn (N.J.); 1455421950@qq.com (Z.H.)

2 Key Laboratory for Molecular Enzymology and Engineering of the Ministry of Education, College of Life Sciences, Jilin University, Changchun 130012, China; zhangchuang19@mails.jlu.edu.cn (C.Z.); ls2012@jlu.edu.cn(S.L.); lzq@jlu.edu.cn(Z.L.)

3 State Key Laboratory of Supramolecular Structure and Materials, College of Chemistry, Jilin University, Changchun, Jilin 130012, China; lmeng17@mails.jlu.edu.cn (M.L.)

4 Key Laboratory of Straw Biology and Utilization, The Ministry of Education, College of Life Science, Jilin Agricultural University, Changchun 130118, China

* Correspondence: wzf@jlau.edu.cn (Z.W.); lc2018@jlu.edu.cn (C.L.); Tel.: +86-431-84532857 (Z.W.); +86-431-87836710 (C.L.)

[#] These authors contributed equally to this work.

Electronic Supplementary Information

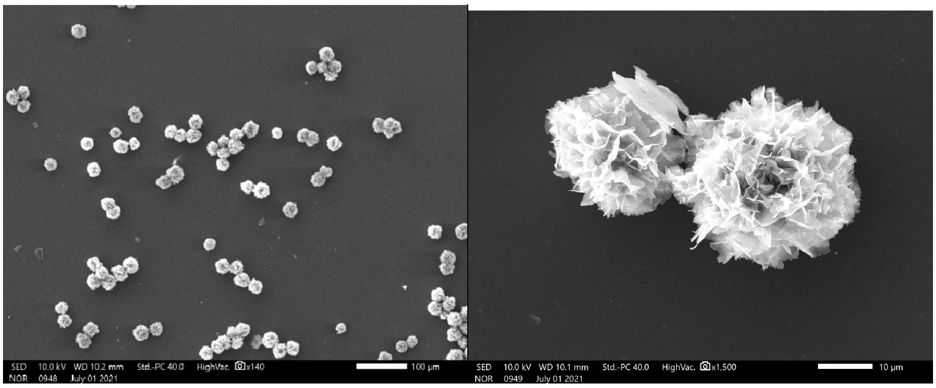

（a） （b）

(1) D-Alanine-incorporated nanoflower

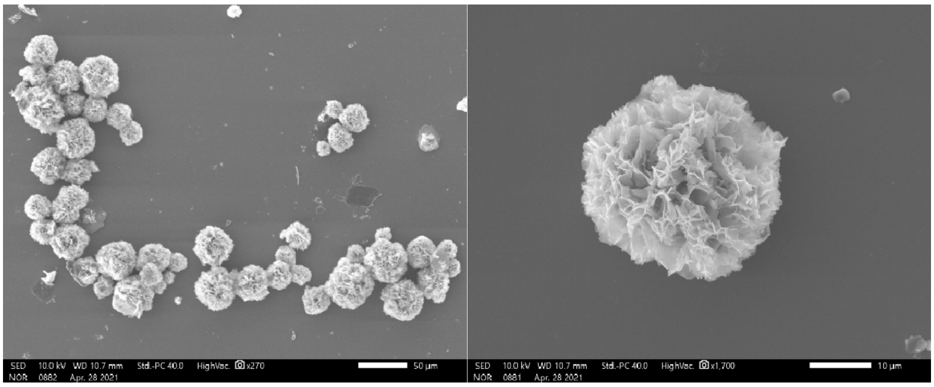

(a) (b)

(2) D-Aspartic acid-incorporated nanoflower

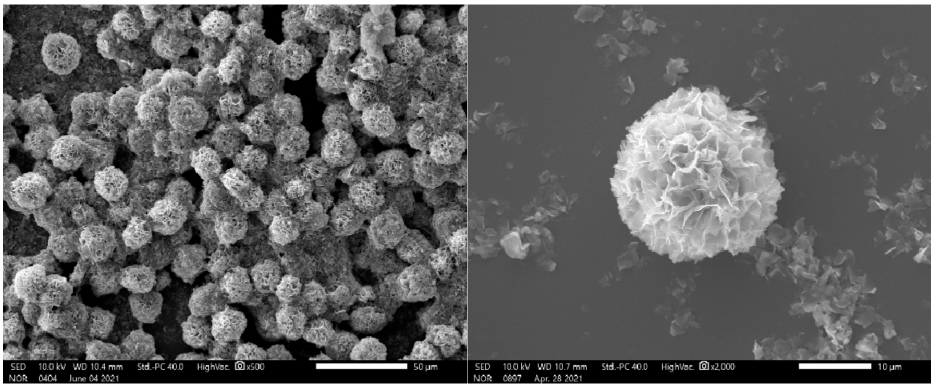

(a) (b)

(3) D-Asparagine acid-incorporated nanoflower

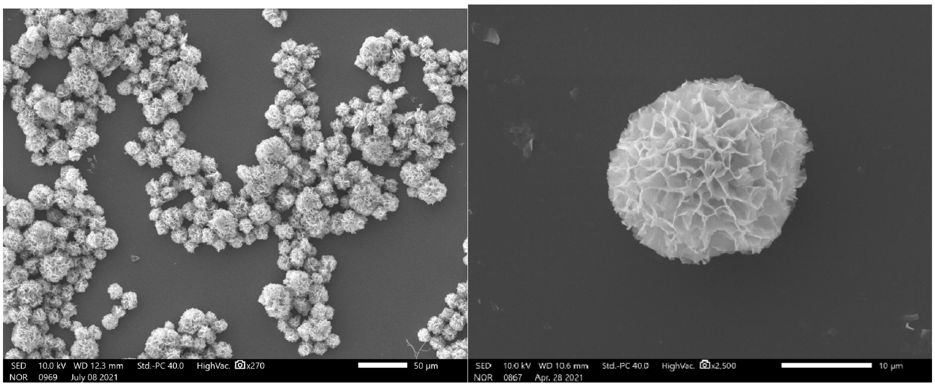

(a) (b)

(4) D-Argnine-incorporated nanoflower

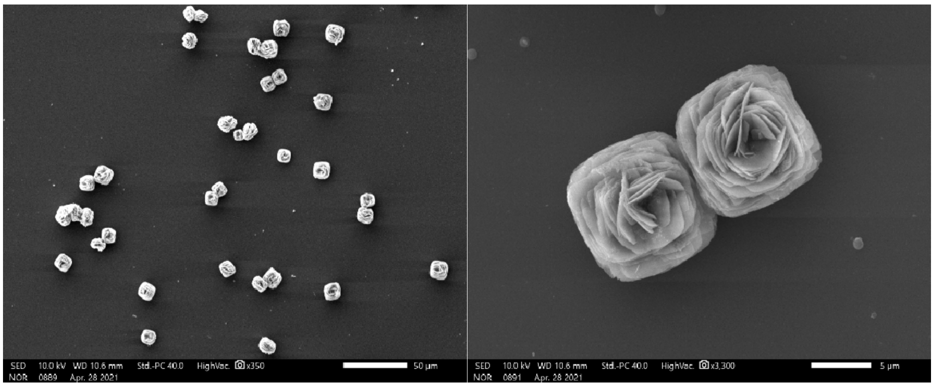

(a) (b)

(5) D-Cysteine-incorporated nanoflower

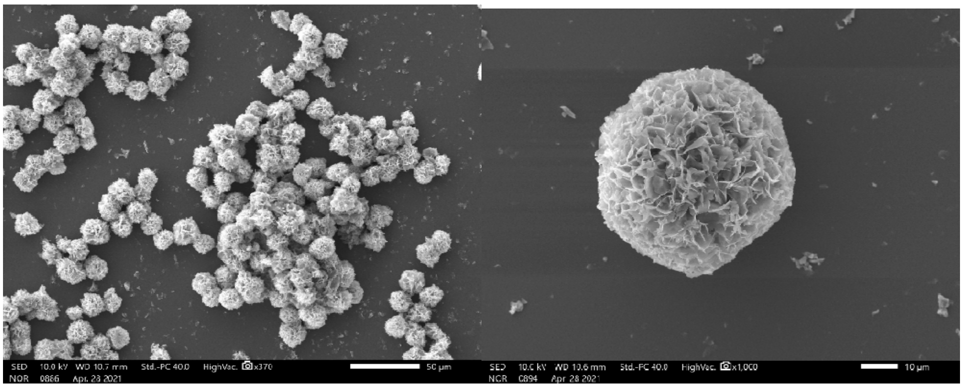

(a) (b)

(6) D-Glutamine-incorporated nanoflower

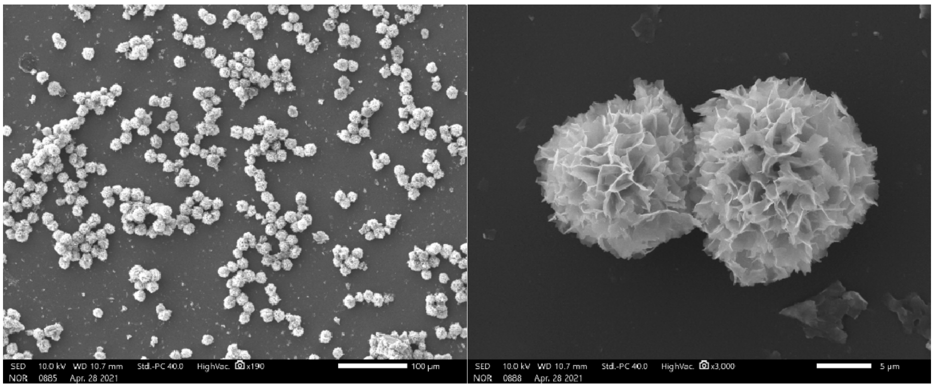

(a) (b)

(7) D-Glutamic acid-incorporated nanoflower

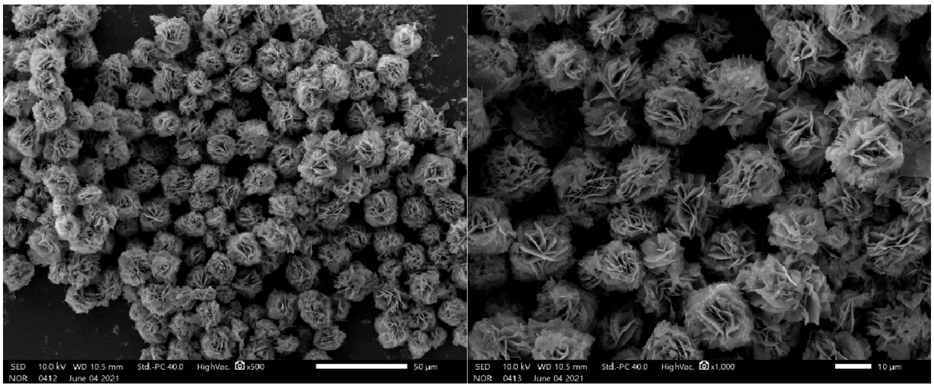

(a) (b)

(8) D-Histidine-incorporated nanoflower

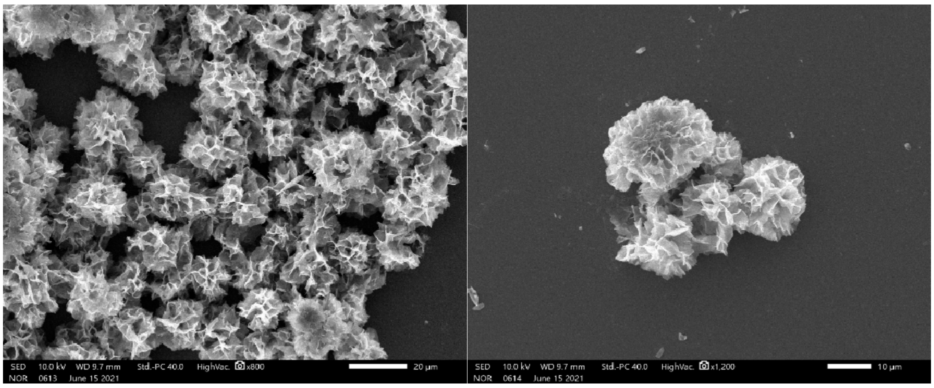

(a) (b)

(9) D-Leucine-incorporated nanoflower

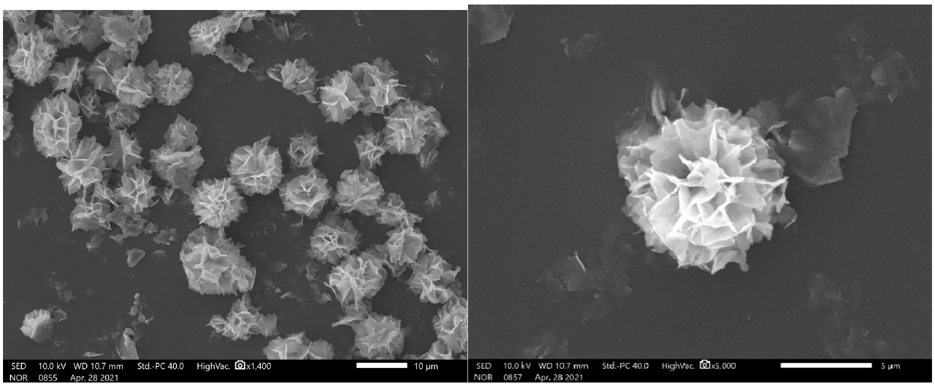

(a) (b)

(10) D-Lysine-incorporated nanoflower

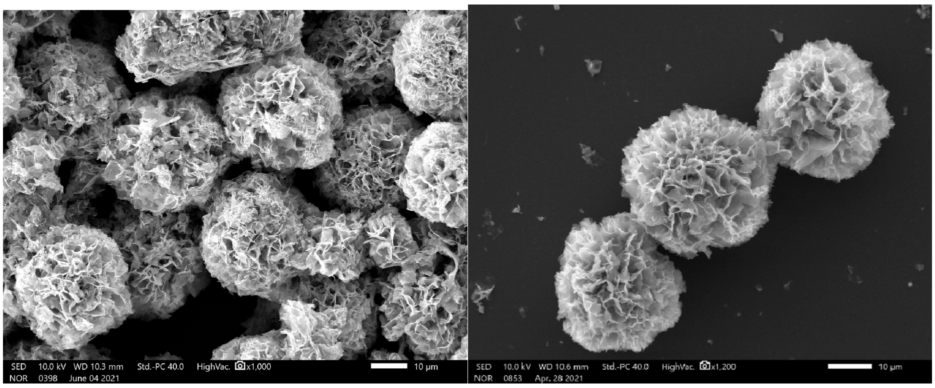

(a) (b)

(11) D-Methionine-incorporated nanoflower

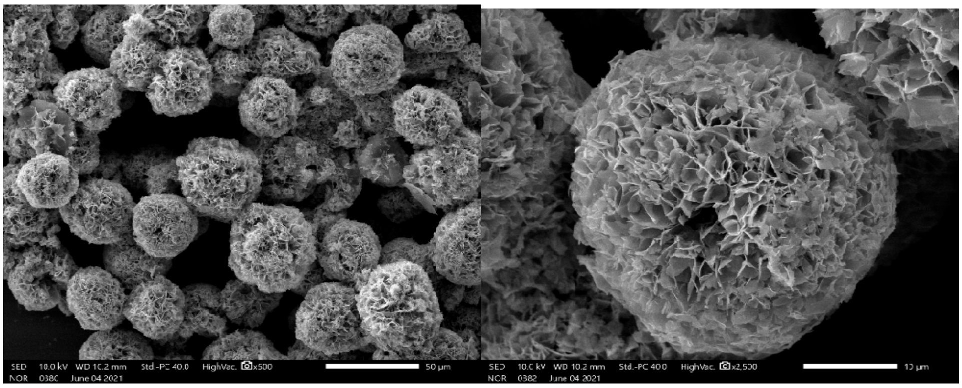

(a) (b)

(l2) D-Serine-incorporated nanoflower

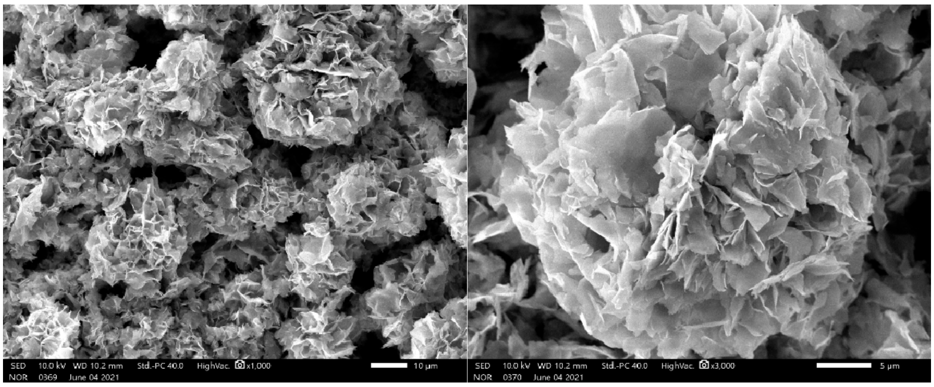

(a) (b)

(13) D-Proline-incorporated nanoflower

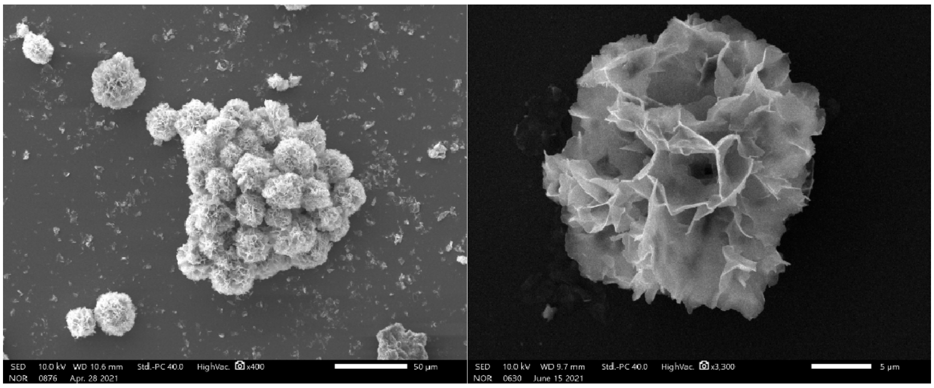

(a) (b)

(14) D-Phenylalanine-incorporated nanflower

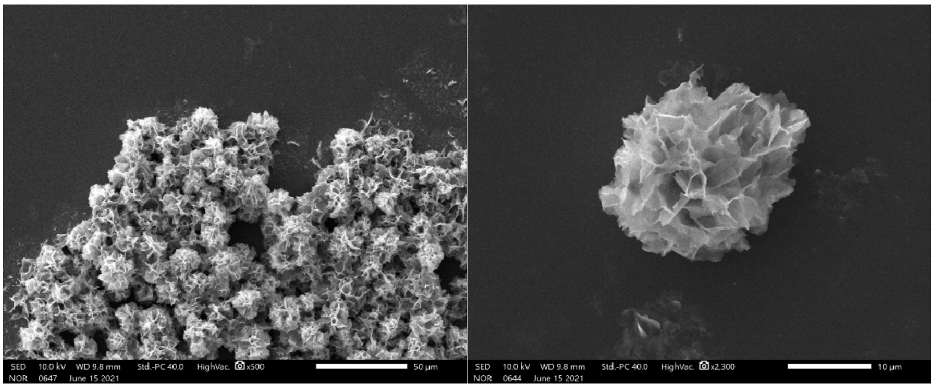

(a) (b)

(15) D-Tryptophane-incorporated nanoflower

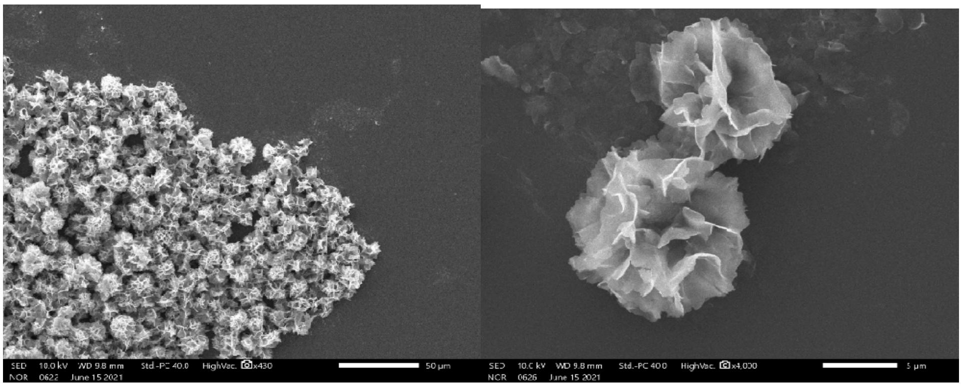

(a) (b)

(16) D-Threonine-incorporated nanoflower

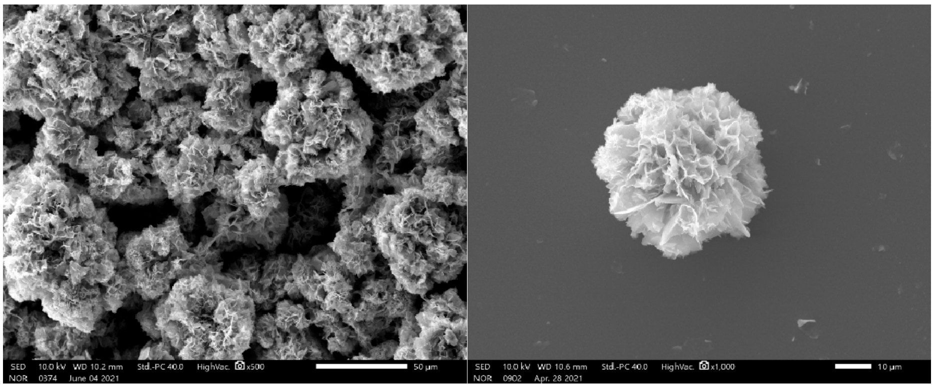

(a) (b)

(17) D-Tyrosine-incorporated nanoflower

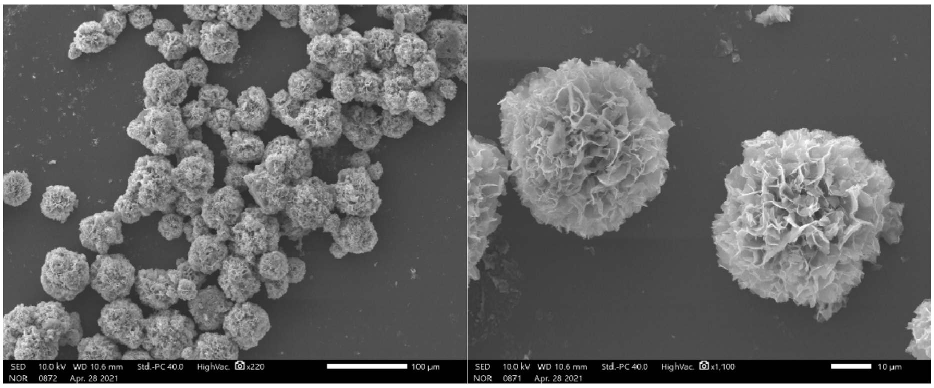

(a) (b)

(18) D-Valine-incorporated nanoflower

Figure S1. SEM images of different amino acid-incorporated nanoflowers (1-18): (1) D-Alanine, (2) D-Aspartic acid, (3) D-Asparagine acid, (4) D-Argnine, (5) D-Cysteine, (6) D-Glutamine, (7) D-Glutamic acid, (8) D-Histidine, (9) D-Leucine, (10) D-Lysine, (11) D-Methionine, (12) D-Serine, (13) D-Proline, (14) D-Phenylalanine, (15) D-Tryptophane, (16) D-Threonine, (17) D-Tyrosine and (18) D-Valine.

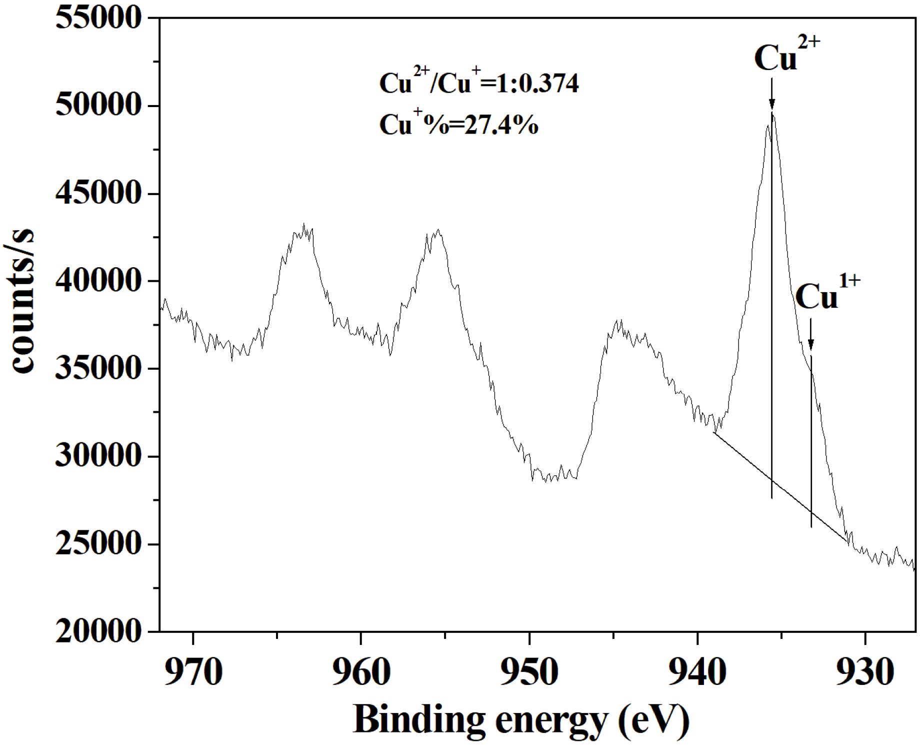

Figure S2. XPS spectrum of ILE incorporated nanoflower.

Table S1. The comparison in linear range and limit of detection between different detection systems for detecting H_2O_2

Catalyst	Linear range (μM)	LOD (μM)	Reference
The nanoflowers	10-700	9.51	This work
Au/Co_3O_4-CeOx NCs	10–100	5.29	[1]
FePt-Au HNPs	20–700	12.33	[2]
Cu-Ag/rGO	1-30	3.82	[3]
MMT-CeO_2 NPs	9–500	7.8	[4]
Fe-GDY/GOx	5-160	0.89	[5]
N-G-Fe_3O_4	0–10	17.1	[6]
$CuMnO_2$	25-300	11.26	[7]
Graphene–AuNPs	20–280	6	[8]
CoS	50–800	20	[9]
NiFe-LDHNS	10-500	4.4	[10]

Catalyst	Linear range (μM)	LOD (μM)	Reference
The nanoflowers	1-30	0.096	This work
g-C_3N_4/MnO_2	0.2-100	0.05	[11]
V_2O_5	0.01-0.5	0.024	[12]
Fe_3O_4/CNDs	0.1–20	0.058	[13]
Au NPs	1-40	0.013	[14]

Co_3O_4-MMT	0.1-20	0.088	[15]
MnO_2/CDs	0.1-10	0.095	[16]
PSMOF	0-20	0.68	[17]
$FeMnO_3$	0-10	0.036	[18]
SPB-MnO_2	Not given	0.45	[19]

Table S2. The comparison in linear range and limit of detection between different detection systems for detecting GSH

Reference

1. Liu, H.; Ding, Y.; Yang, B.; Liu, Z.; Liu, Q.; Zhang, X. Colorimetric and ultrasensitive detection of H_2O_2 based on Au/Co_3O_4-CeOx nanocomposites with enhanced peroxidase-like performance. *Sens. Actuators, B* **2018**, *271*, 336-345, doi:10.1016/j.snb.2018.05.108

2. Ding, Y.; Yang, B.; Liu, H.; Liu, Z.; Zhang, X.; Zheng, X.; Liu, Q. FePt-Au ternary metallic nanoparticles with the enhanced peroxidase-like activity for ultrafast colorimetric detection of H_2O_2. *Sens. Actuators, B* **2018**, *259*, 775-783, doi:10.1016/j.snb.2017.12.115

3. Darabdhara, G.; Sharma, B.; Das, M.R.; Boukherroub, R.; Szunerits, S. Cu-Ag bimetallic nanoparticles on reduced graphene oxide nanosheets as peroxidase mimic for glucose and ascorbic acid detection. *Sens. Actuators, B* **2017**, *238*, 842-851, doi:10.1016/j.snb.2016.07.106

4. Sun, L.; Ding, Y.; Jiang, Y.; Liu, Q. Montmorillonite-loaded ceria nanocomposites with superior peroxidase-like activity for rapid colorimetric detection of H_2O_2. *Sens. Actuators, B* **2017**, *239*, 848-856, doi:10.1016/j.snb.2016.08.094

5. Liu, J.M.; Shen, X.M.; Baimanov, D.; Wang, L.M.; Xiao, Y.T.; Liu, H.B.; Li, Y.L.; Gao, X.F.; Zhao, Y.L.; Chen, C.Y. Immobilized Ferrous Ion and Glucose Oxidase on Graphdiyne and Its Application on One-Step Glucose Detection. *ACS Appl. Mater. Interfaces* **2019**, *11*, 2647-2654, doi:10.1021/acsami.8b03118.

6. Zhang, W.J.; Chen, C.P.; Yang, D.X.; Dong, G.X.; Jia, S.J.; Zhao, B.X.; Yan, L.; Yao, Q.Q.; Sunna, A.; Liu, Y. Optical Biosensors Based on Nitrogen-Doped Graphene Functionalized with Magnetic Nanoparticles. *Adv. Mater. Interfaces* **2016**, *3*, 5, doi:10.1002/admi.201600590.

7. Chen, Y.; Chen, T.; Wu, X.; Yang, G. $CuMnO_2$ nanoflakes as pH-switchable catalysts with multiple enzyme-like activities for cysteine detection. *Sens. Actuators, B* **2019**, *279*, 374-384,

doi:10.1016/j.snb.2018.09.120

8. Hu, J.; Li, F.; Wang, K.; Han, D.; Zhang, Q.; Yuan, J.; Niu, L. One-step synthesis of graphene–AuNPs by HMTA and the electrocatalytical application for O_2 and H_2O_2. *Talanta* **2012**, *93*, 345-349, doi:10.1016/j.talanta.2012.02.050

9. Yang, H.; Zha, J.; Zhang, P.; Xiong, Y.; Su, L.; Ye, F. Sphere-like CoS with nanostructures as peroxidase mimics for colorimetric determination of H_2O_2 and mercury ions. *RSC Adv.* **2016**, *6*, 66963-66970, doi:10.1039/c6ra16619a

10. Zhan, T.; Kang, J.; Li, X.; Pan, L.; Li, G.; Hou, W. NiFe layered double hydroxide nanosheets as an efficiently mimic enzyme for colorimetric determination of glucose and H_2O_2. *Sens. Actuators, B* **2018**, *255*, 2635-2642, doi:10.1016/j.snb.2017.09.074

11. Fu, X.-L.; Hou, F.; Liu, F.-R.; Ren, S.-W.; Cao, J.-T.; Liu, Y.-M. Electrochemiluminescence energy resonance transfer in 2D/2D heterostructured g-C_3N_4/MnO_2 for glutathione detection. *Biosens. Bioelectron.* **2019**, *129*, 72-78, doi:10.1016/j.bios.2019.01.010

12. Ganganboina, A.B.; Doong, R.-a. The biomimic oxidase activity of layered V_2O_5 nanozyme for rapid and sensitive nanomolar detection of glutathione. *Sens. Actuators, B* **2018**, *273*, 1179-1186, doi:10.1016/j.snb.2018.07.038

13. Luo, N.; Yang, Z.; Tang, F.; Wang, D.; Feng, M.; Liao, X.; Yang, X. Fe_3O_4/carbon nanodot hybrid nanoparticles for the indirect colorimetric detection of glutathione. *ACS Appl. Nano Mater.* **2019**, *2*, 3951-3959, doi:10.1021/acsanm.9b00854

14. Kumar, V.; Bano, D.; Singh, D.K.; Mohan, S.; Singh, V.K.; Hasan, S.H. Size-dependent synthesis of gold nanoparticles and their peroxidase-like activity for the colorimetric detection of glutathione from human blood serum. *ACS Sustainable Chem. Eng.* **2018**, *6*, 7662-7675, doi:10.1021/acssuschemeng.8b00503.s001

15. Gao, Y.; Wu, K.; Li, H.; Chen, W.; Fu, M.; Yue, K.; Zhu, X.; Liu, Q. Glutathione detection based on peroxidase-like activity of Co_3O_4–Montmorillonite nanocomposites. *Sens. Actuators, B* **2018**, *273*, 1635-1639, doi:10.1016/j.snb.2018.07.091

16. Wang, Q.; Pang, H.C.; Dong, Y.Q.; Chi, Y.W.; Fu, F.F. Colorimetric determination of glutathione by using a nanohybrid composed of manganese dioxide and carbon dots. *Microchim. Acta* **2018**, *185*, 7, doi:10.1007/s00604-018-2830-6.

17. Liu, Y.; Zhou, M.; Cao, W.; Wang, X.; Wang, Q.; Li, S.; Wei, H. Light-Responsive Metal–Organic Framework as an Oxidase Mimic for Cellular Glutathione Detection. *Anal. Chem.* **2019**, *91*, 8170-8175, doi:10.1021/acs.analchem.9b00512.

18. Chi, M.; Chen, S.; Zhong, M.; Wang, C.; Lu, X. Self-templated fabrication of $FeMnO_3$ nanoparticle-filled polypyrrole nanotubes for peroxidase mimicking with a synergistic effect and their sensitive colorimetric detection of glutathione. *Chem. Commun.* **2018**, *54*, 5827-5830, doi:10.1039/c8cc01574k

19. Yang, Q.; Li, L.; Zhao, F.; Wang, Y.; Ye, Z.; Guo, X. Generation of MnO_2 nanozyme in spherical polyelectrolyte brush for colorimetric detection of glutathione. *Mater. Lett.* **2019**, *248*, 89-92, doi:10.1016/j.matlet.2019.04.007
